# Novel Polyethylene Glycol-Conjugated Triazole Derivative with High Thyrointegrin αvβ3 Affinity in Acute Myeloid Leukemia Management

**DOI:** 10.3390/cancers13164070

**Published:** 2021-08-13

**Authors:** Thangirala Sudha, Kavitha Godugu, Noureldien H. E. Darwish, Tipu Nazeer, Shaker A. Mousa

**Affiliations:** 1 Pharmaceutical Research Institute, Albany College of Pharmacy and Health Sciences, Rensselaer, NY 12144, USA; sudha.thangirala@acphs.edu (T.S.); Kavitha.Godugu@acphs.edu (K.G.); noureldien.darwish@acphs.edu (N.H.E.D.); 2 Hematology Unit, Clinical Pathology Department, Faculty of Medicine, Mansoura University, Mansoura 35516, Egypt; 3 Albany Medical Center, Pathology Department, AMC Hospital, Albany, NY 12208, USA; NazeerT@amc.edu

**Keywords:** thyrointegrin receptor, αvβ3, P-bi-TAT, leukemia, AML therapy

## Abstract

**Simple Summary:**

The integrin αvβ3 antagonist (P-bi-TAT) is associated with the downregulation of signaling pathways, especially NF-κB pathway, which play an important role in leukemogenesis. Our results provide a rationale for further evaluation of the αvβ3 antagonists as providing potential therapeutic utility not only in AML but also in different malignancies.

**Abstract:**

(1) Background: Acute myeloid leukemia (AML) accounts for up to one-third of more than 60,000 leukemia cases diagnosed annually in the U.S. Primary AML cells express membrane αvβ3 integrin, which is associated with adverse prognosis and resistance to chemotherapies. A novel anticancer compound Polyethylene glycol-conjugated bi-TriAzole Tetraiodothyroacetic acid (P-bi-TAT) interacts with high affinity (Ki 0.3 nM) and specificity with the thyrointegrin αvβ3. We evaluated P-bi-TAT activities in two different AML models representing monocytic and myelocytic forms of acute leukemia. (2) Methods and Results: The in vivo AML models were established prior to initiation of treatment protocols by grafting human leukemia cells in immunocompromised mice. IVIS imaging scans revealed that leukemic colonies were extensively established throughout the bone marrow, liver, and lung of the untreated animals. In animals treated with P-bi-TAT at daily doses ranging from 1–10 mg/kg, subcutaneously for 2–3 weeks, IVIS imaging scans revealed 95% reduction in bone marrow colonies and leukemic colonies in liver and lung. Also, the leukemic cells were not detected in bone marrow samples of P-bi-TAT-treated animals. The anti-neoplastic effect of P-bi-TAT administration on leukemic cells was associated with marked inhibition of NF-κB activity. We conclude that experimental P-bi-TAT therapy in vivo appears extraordinarily effective against the two forms of human AML models in mice. Because the P-bi-TAT molecular target, thyrointegrin αvβ3, is consistently expressed in many, if not all, clinical AML samples, P-bi-TAT-based therapy seems to have significant clinical potential in treating most AML sub-types. Hence, P-bi-TAT represents a promising targeted therapeutic agent for AML patients.

## 1. Introduction

The estimated overall incidence of leukemia in the United States for 2020 is about 60,000, with a death rate of about 38% (23,100 deaths from all leukemias). Acute myeloid leukemia (AML) represents one third of new cases, with a death rate of about 56% (11,000 deaths from AML) [1]. AML is generally uncommon before the age of 45, and the average age of people first diagnosed with AML is about 65 years [2]. Despite achievements in improving survival of patients diagnosed with AML, especially younger patients, overall AML long-term survival is still an area of significant clinical need and an important focus of active research [3,4,5]. Previous studies indicate that integrins are playing a key role in promoting tumor cell survival and invasion, suggesting that therapeutic targeting of the functions of some integrins, such as αvβ3, which has thyroid hormone analogue receptors and it referred to here as thyrointegrin, may suppress the activity of the most aggressive and metastatic cancer cells in AML, breast, and pancreatic malignancies [5,6,7]. Recent studies from our laboratories documented the key role of thyrointegrin αvβ3 in the immune modulation and other crucial biological features of various types of human cancers [8,9].

The integrin family of cell surface macromolecules is a group of transmembrane receptors that bind adhesion receptors on neighboring cells or extracellular matrix (ECM) proteins and regulate a plethora of different cellular functions. The specificity of integrin binding to one or more cognate ligands depends mainly on the heterodimeric pairing of integrin α and β subunits [10,11]. Some heterodimeric pairings occur preferentially with different types of ligands: αvβ3 binds vitronectin, fibrinogen, fibronectin, ostepontin, thrombospondin, vWF, fibrillin, and tenascin [12,13]. The binding affinity between integrins and ligands plays an important role in the regulation and control of the functions of endothelial cells during angiogenesis, localization of inflammatory cells recruited to sites of injury and repair, or the invasive potential of tumor cells [14].

The thyrointegrin αvβ3 mediates intravasation and extravasation of tumor cells and has been implicated in the malignant spread of tumor cell types such as melanoma, colon cancer, and gliomas [15,16]. Thus, this cell surface receptor is an attractive target for cancer therapy, particularly if there is a molecularly defined opportunity for refinement of targeting to discrete domains of the protein that does not involve the interference with desirable physiological functions of αvβ3 molecules [17,18].

Here, we focused on a novel nano-pharmaceutical compound named P-bi-TAT comprised of Polyethylene glycol (PEG) covalently bonded to two Tri-Azole Tetraiodothyroacetic acid molecules (P-bi-TAT). P-bi-TAT is designed to optimize the potency and broaden the anticancer properties of cancer therapeutics designated as thyrointegrin αvβ3 signaling antagonists [19,20]. Furthermore, NF-κB signal transduction pathway represents one of the key signaling pathways, the activation of which is consistently implicated in the growth and survival of human AML cells [21]. Therefore, we carried out the luciferase activity assay specifically to monitor the NF-κB pathway activity in human AML cells exposed to various concentrations of P-bi-TAT. P-bi-TAT acts on target cells without nuclear translocation and has demonstrated effective suppression of cancer cell proliferation, survival, and invasion, as well as molecular interference with expression of genes essential for growth and survival of cancer cells [19,20].

## 2. Materials and Methods

### 2.1. Tumor Cells and Test Compound

AML cell line (Kasumi-1 cells, myelocytic leukemia) was from American Type Culture Collection (ATCC, Manassas, VA, USA). THP1-lucia™ cell line (human monocytic cell line derived from an acute monocytic leukemia patient) was from InvivoGen (San Diego, CA, USA). These cell lines are commonly used in research studies as AML cell lines [22,23].

The Kasumi-1 cell line is an intensively investigated model system of AML with t (8;21) translocation that results in a fusion protein (AML1-ETO), which plays an important role in the downregulation of CEBPA mRNA and protein and DNA binding activity, leading to great disturbance in granulocytic differentiation [24,25]. THP-1 cells represent the t (9;11), which is associated with acute monocytic leukemia [26].

P-bi-TAT was synthesized at the Pharmaceutical Research Institute (Rensselaer, NY, USA) [19].

### 2.2. In Vitro Studies

(a) Evaluation of NF-κB Activity

We analyzed the activity of the NF-κB signal transduction pathway using THP1-lucia cell line. THP1-lucia is derived from the human THP-1 monocyte cell line by stable integration of an NF-κB-inducible Luc reporter construct designed to monitor the NF-κB signaling pathway activity. THP1-lucia cells allow the monitoring of NF-κB activity status by measuring the activity of secreted luciferase (Lucia). Cells were incubated with various concentrations of P-bi-TAT (0.2, 1, 2, 10, 20, and 40 µM). After 48 h, the supernatant was collected and assessed with luciferase reagent (Quanti1-luc, InvivoGen). Luciferase reagent was added to the collected supernatant samples, and the luminescence was measured with the GloMax Luminometer (Promega Corporation, Madison, WI, USA).

(b) Cell Vitality Assay (MTT Assay)

The cell vitality assay was carried out on the two human AML cell lines. Cells were seeded in 96-well plates (50 × 10^3^–100 × 10^3^ per well) and were treated with the different concentrations of P-bi-TAT (0.2–40 µM). The cellular viability of suspended cells was determined using the MTT assay. All reactions were performed in triplicate. Measured data of cellular viability were normalized using viability values of untreated control cells (100%).

### 2.3. In Vivo Studies

Animal studies were carried out at the Albany Stratton VA Medical Center (Albany, NY, USA) animal facility, and protocols were approved by the VA IACUC. Mice were used in compliance with Public Health Service Policy on Humane Care and Use of Laboratory Animals. We established the in vivo human AML animal models using NOD/SCID mice. Seventy mice were purchased from Taconic Bioscience Breeding Laboratories (Rensselaer, NY, USA). Mice were housed under specific pathogen-free conditions and controlled room temperature conditions (20–24 °C) and humidity (60–70%). Mice were acclimatized for 1 week prior to the experimental treatment.

Several routes of injections of leukemic cells were employed, including intraperitoneal (i.p.) and intravenous (i.v.) routes [27,28,29]. Zhang et al., reported the successful engraftment of leukemic cells by i.p. route [30]. We established our AML models by i.p injection and the treatment was started after confirmation of successful engraftment using IVIS, peripheral blood smear, and bone marrow examination. The 70 NOD/SCID mice were divided into two main groups; one group (40 mice; 20 males and 20 females) was injected intraperitoneally with 10–20 ×10^6^ with Kasumi-1cells (human myeloid leukemia model) and the other group (30 mice; 15 males and 15 females) injected with THP-1-Luc (human monocytic leukemia model).

Prior to implantation, the NOD/SCID mice were pretreated with Cyclophosphamide (20 mg/kg) about 24 h before the intraperitoneal injection of leukemic cells. Cyclophosphamide was used to decrease the possibility of rejection of human cells by the mice and to improve engraftment of the leukemic cells in the mouse bone marrow.

We evaluated P-bi-TAT in three mice subgroups (1, 3, and 10 mg/kg) in comparison to a control group (PBS) (*n* = 4–5 mice/subgroup). P-bi-TAT at specified doses (experimental groups) or PBS (control groups) was injected daily subcutaneously for 3 weeks beginning at day 21 after the inoculation of the AML cells. In vivo imaging scans (IVIS), peripheral blood samples, and smears of bone marrow were used to evaluate the disease status in animals. At termination of the therapy protocols, mice were humanely sacrificed and processed to obtain samples of peripheral blood smears, bone marrow aspirates, and samples of multiple internal organs for histological examinations.

### 2.4. Statistical Analysis

Results are presented as means ± S.E.M. or ± S.D. as indicated in Figure legends. Statistical analysis was performed using one-way ANOVA followed by Newman–Keuls post-test when more than two groups were being compared. Differences between groups were considered statistically significant for *p* ≤ 0.01. All statistical analyses were performed using GraphPad InStat 3 (GraphPad, San Diego, CA, USA).

## 3. Results

### 3.1. In Vitro Study of P-bi-TAT Effect on Cell Viability and NF-κB Pathway Activity

**Figure 1:**
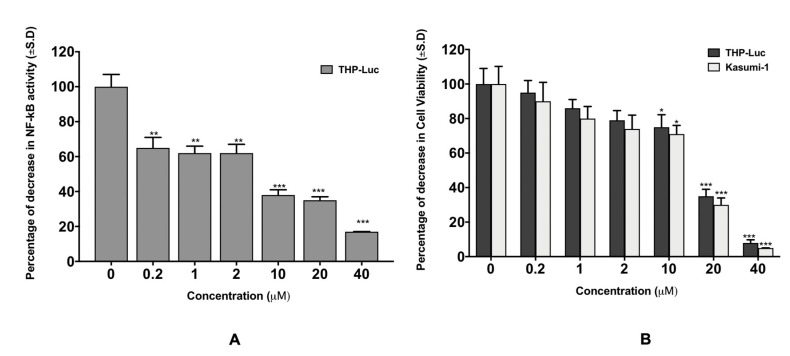
P-bi-TAT effect on leukemic cell lines. (**A**) THP1-lucia NF-kB activity (luciferase assay) after 48-h treatment, (**B**) THP1-lucia and Kasumi-1 cell vitality (MTT assay) after 48-h treatment. Results are expressed as mean ± S.D., *n* = 3. (*** *p* < 0.0001, ** *p* < 0.001, * *p* < 0.01).

We carried out several in vitro experiments designed to explore the potential mechanisms of the anticancer activity in vivo of the novel thyrointegrin antagonist P-bi-TAT. The in vitro experiments were performed in triplicate and repeated three time using the human AML cell lines THP-1 and Kaumi-1. In the 48-h MTT assay, THP-1 and Kaumi-1 treated with 0.2–40 µM P-bi-TAT manifested a significant dose-dependent decrease in cell viability in comparison to control cultures (Figure 1A).

These experiments showed that P-bi-TAT treatments at different concentrations demonstrated a concentration-dependent decrease in NF-κB activity in THP-1-Luc cells (from 40% to 90% inhibition). Furthermore, the treatment of human leukemic cells with 10 µM P-bi-TAT (the dose that manifested marked anticancer activity in vivo) was associated with ~65% decrease in NF-κB activity (*p* < 0.0001) (Figure 1B).

### 3.2. In Vivo Study of P-bi-TAT Therapy Efficacy

P-bi-TAT was injected daily for 21 days and mice body weight was monitored throughout the study. There were no changes observed for body weight during the study.

**Figure 2:**
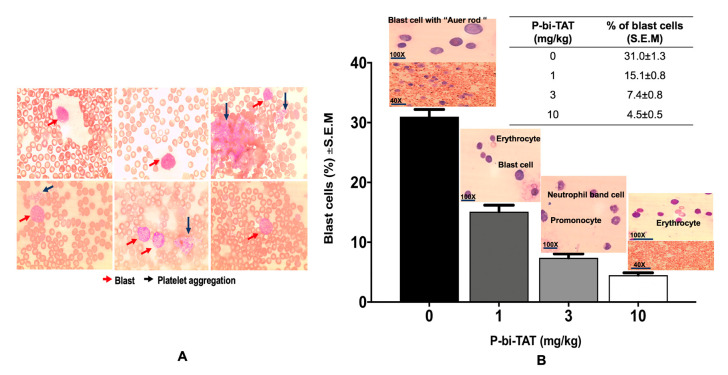
Evaluation of blast cells in peripheral blood smear. (**A**) Blast cells in the blood smears of NOD/SCID mice injected with THP1-lucia after 16 days. Red arrow, blast cells; Black arrow, platelets aggregation. (**B**) Blood smear with Lishman stain represent AML control blood smears vs. P-bi-TAT-treated animal cells. Control group shows many blast cells in the peripheral blood, and P-bi-TAT (3 mg/kg) treated animal shows no blast cells. Results are expressed as mean ± S.D., *n* = 4–5 mice/group.

The leukemic cells could be readily detected in the peripheral blood smear on day 10 for all mice. With the progression of the disease, leukemic cells were high in number in the blood smear of the control group, while no blast cells could be detected in the treated group (Figure 2).

**Figure 3:**
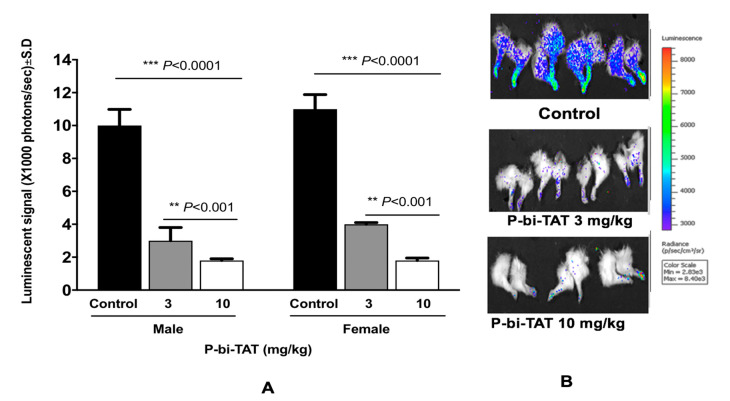
P-bi-TAT effect on bone marrow metastasis in AML mice as evaluated with In Vivo Imaging System (IVIS). (**A**) High luminescent signal intensity was observed in the control group of mice in comparison to the treated groups. No differences in signal intensity could be detected between males and females. (**B**) Bioluminescent signals of THP1-lucia cells following termination in mouse bone marrow (showing hind legs). Results showed a successful decrease of engraftment of the leukemic cells in the group treated with P-bi-TAT (10 mg/kg). The color bar shows the relative color for signal intensity, ranging from nonviable (**blue**) to fully viable (**red**). (*** *p* < 0.0001, ** *p* < 0.001). Results are expressed as mean ± S.D., *n* = 4–5 mice/group.

**Figure 4:**
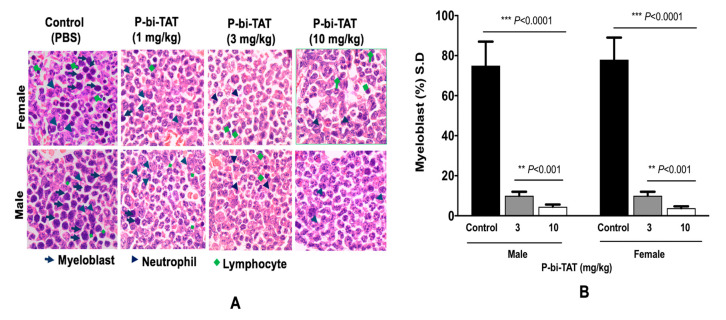
Myeloblast in bone marrow of NOD/SCID mice after 21 days of treatment with P-bi-TAT (3, and 10 mg/kg) (**A**) Blast cells decreased ~75–85% in comparison to the control group. (**B**) Percentage of myeloblast in bone marrow. Results are expressed as mean ± S.D., *n* = 4–5 mice/group, *** *p* < 0.001, ** *p* < 0.0001.

IVIS scans for the THP-1 Luc group showed marked reduction of engraftment of leukemic cells in bone marrow (~95%) in animals treated with 10 µM of P-bi-TAT daily, *p* < 0.0001 (Figure 3), which was confirmed with histopathology results (Figure 4). Also, there were no detected metastases in liver, kidney, or lung in animals treated with P-bi-TAT in comparison to the control group.

On termination of the experimental therapy protocol, peripheral blood and tissues samples were collected from euthanized mice for postmortem examinations, including bone marrow, spleen, lung, and liver samples. Normal myeloid precursors, which are the source of functional white blood cells, were found in the marrow smears, but leukemic cells were not detected in P-bi-TAT-treated animals. The leukemic cells in the bone marrow of all mice were decreased by 50%, 75%, and 85% with P-bi-TAT treatment at 1, 3, and 10 mg/kg, respectively. On the other hand, marked infiltration with leukemic cells was observed in the bone marrows of all animals in the control group (~100%) (Figure 4).

## 4. Discussion

AML is a complex aggressive hematological malignancy that affects the blood and lymphoid system of the body [31]. AML is characterized by abnormal proliferation of immature blast cells in bone marrow, spreading into the blood stream and other organs e.g., lymph nodes, spleen, and liver. The 5-year survival rate for AML is less than 24% [32], although recently there have been more selective and targeted immunotherapy options developed [33,34,35] that have slightly increased the 5-year survival rate. These new therapies are either used alone or in combination with chemotherapy to achieve a better outcome. The adverse effects of these therapies can be challenging, and more research is needed on how to optimize the therapy for the best possible outcome [36,37].

Recent clinical studies have demonstrated that heterodimeric integrin cell surface molecules composed of α and β chains, which can bind ECM molecules, cell surface molecules, and variable soluble mediators [38,39], have a role in AML progression and cessation. This has raised interest to develop therapeutic agents targeting integrin receptors. The β3 integrin (ITGB3) chain can form heterodimers only with the two α chains, αIIb and αV, which play an important role in leukemogenesis and chemo-resistance in human AML [40,41].

Both subunits (α and β) are transmembrane proteins containing large extracellular domains, a single transmembrane domain, and a small cytoplasmic tail. Integrins play an important role in both the assembly of both cytoskeletal polymers and intracellular signaling complexes [42]. Thyrointegrin αvβ3 binds to ECM molecules or counter-receptors on neighboring cells, e.g., AML cells with the ECM or neighboring nonleukemic cells [43,44,45].

A recent study reported that ITGB3 expression is higher in older AML patients (median age, 57.8; *p* < 0.05), especially in those with unfavorable cytogenetics (*p* = 0.002), e.g., Fms-like receptor tyrosine kinase 3 (FLT3)-internal tandem duplications (ITD) [46]. The expression level of ITGB3 was significantly higher in the poor group than in favorable and intermediate AML cytogenetic abnormalities-associated groups [46].

β3 integrins play an important role in activation of several downstream nonreceptor protein tyrosine kinases, e.g., spleen tyrosine kinase (Syk) and focal adhesion kinase (FAK) [47,48]. Syk activation seems to play an important role for the effects of ITGB3 on AML cell homing, transcriptional regulation in leukemic stem cells, and differentiation induction of the leukemic cells [46]. This was clearly demonstrated by an experimental study of a megakaryoblast leukemic cell line that showed that combined soluble and solid phase fibrinogen exposure caused tyrosine phosphorylation of the β3 and at the same time complex formation with Syk; the additional translocation of Syk to the cytoskeleton looks to be a two-step process and one of these late steps is also β3 dependent [48].

Several studies have reported the role of FAK in the regulation of proliferation, migration, and chemoresistance of human AML cells [49,50,51,52,53]. One of these studies reported that inhibition of FAK suppresses the constitutive growth of the FLT3–and KIT-bearing cells by ~50% at 8 µM of FAK antagonist. Even though these studies suggest that the functional interactions between upstream β3 integrins and FAK have a clinical relevance, further studies are necessary to clarify the potential importance of β3 integrins-initiated signaling for the role of FAK expression and activation in human AML [54]. Observations documented in this study indicated that blocking of the αvβ3 thyrointegrin was associated with a marked decrease in leukemic cell engraftment in bone marrow and multiple organs (>95%), consistent with the hypothesis that thyrointegrin αvβ3 blocking may efficiently interfere with multiple downstream signaling pathways.

Our previous study showed that the anticancer actions of thyrointegrin αvβ3 antagonists were associated with marked inhibition of cisplatin-induced NF-κB activity [55]. Consistently, observations reported in the present study demonstrated that inhibitory effects of P-bi-TAT on growth and survival of human leukemic cells were associated with more than 65% reduction of the NF-κB pathway activity. Also, we reported that P-bi-TAT is acting on the extracellular domain of αvβ3 to regulate, by signal transduction pathways, the expression of a substantial number of genes relevant to cell division, apoptosis, and angiogenesis [56]. Among the genes whose transcription is downregulated by P-bi-TAT are KIT, HRAS, and INH2 [20,56,57,58]. Transcription of pro-apoptotic P53 is upregulated by P-bi-TAT [20,59,60,61].

**Figure 5:**
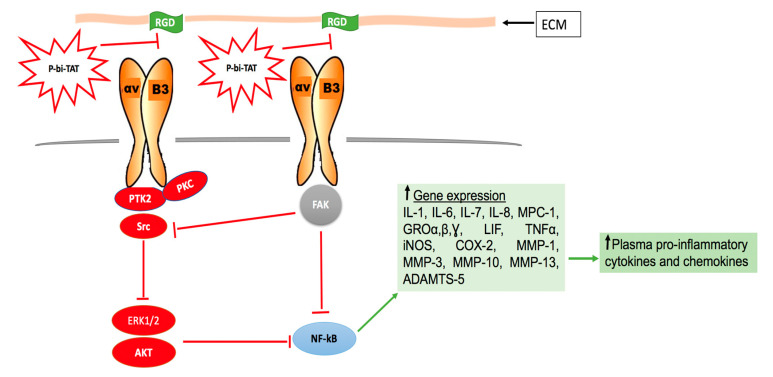
Scheme of the mechanism of P-bi-TAT in blocking thyrointegrin αvβ3 and its impact on NF-kB signaling pathways. Extracellular matrix (ECM) contains the RGD cell binding sequence that can bind to the thyrointegrin αvβ3 and initiate a cell signaling cascade that results in increased expression of pro-inflammatory mediators. P-bi-TAT interferes with the interaction between RGD and thyrointegrin αvβ3, resulting in downregulation of multiple signaling pathways (FAK-Src, ERK, and AKT). P-bi-TAT downregulates NF-kB signaling pathway through activation of FAK signaling pathway. P-bi-TAT interferes with the action of NF-kB, resulting in downregulation of plasma pro-inflammatory cytokines. Red = inhibition, green = activation. Abbreviations: ADAMT = a disintegrin and metalloproteinase with a thrombospondin, RGD = arginyl glycyl aspartic acid, ERK = extracellular-signal-regulated kinase, FAK = focal adhesion kinase, IL = interleukin, MMP = Matrix metalloproteinase, PKC = protein kinase C, PTK2 = protein tyrosine kinase 2, and NF-κB = nuclear factor kappa-light-chain B.

Collectively, experimental observations reported in this contribution demonstrated a significant therapeutic impact of the novel thyrointegrin αvβ3 antagonist P-bi-TAT on human leukemic cell proliferation, survival, and engraftment (Figure 5).

## 5. Conclusions

In this study, we reported that the novel thyrointegrin αvβ3 antagonist P-bi-TAT manifests a significant anticancer activity in vivo in two models of human AML, which is exemplified by marked suppression of the infiltration of leukemic cells into the bones of both male and female transgenic mice. Mechanistically, we demonstrated that the novel thyrointegrin αvβ3 antagonist P-bi-TAT inhibited NF-kB pathway activity in human leukemic cells in a concentration-dependent manner. Results of the present experiments provide a strong rationale for clinical evaluation of the potential therapeutic utility of antagonists of thyrointegrin αvβ3 as a promising approach to AML treatment. Finally, it will be of interest to further investigate whether this novel therapeutic approach can be combined with conventional cytotoxic therapy or other targeted therapies to facilitate an optimal design of future clinical studies of multimodal combination therapy of human AML.

## Data Availability

The data presented in this study are available in this article and on request from the corresponding author (shaker Mousa, email; shaker.mousa@acphs.edu).
